# *DEGS2* polymorphism associated with cognition in schizophrenia is associated with gene expression in brain

**DOI:** 10.1038/tp.2015.45

**Published:** 2015-04-14

**Authors:** K Ohi, G Ursini, M Li, J H Shin, T Ye, Q Chen, R Tao, J E Kleinman, T M Hyde, R Hashimoto, D R Weinberger

**Affiliations:** 1 Department of Clinical Genetics, Lieber Institute for Brain Development, Johns Hopkins Medical Campus, Baltimore, MD, USA; 2 Department of Psychiatry, Osaka University Graduate School of Medicine, Osaka, Japan; 3 Department of Psychiatry, Johns Hopkins University School of Medicine, Baltimore, MD, USA; 4 Department of Neurology, Johns Hopkins University School of Medicine, Baltimore, MD, USA; 5 Molecular Research Center for Children's Mental Development, United Graduate School of Child Development, Osaka University, Osaka, Japan; 6 Department of Neuroscience, Johns Hopkins University School of Medicine, Baltimore, MD, USA; 7 Institute of Genetic Medicine, Johns Hopkins University School of Medicine, Baltimore, MD, USA

## Abstract

A genome-wide association study of cognitive deficits in patients with schizophrenia in Japan found association with a missense genetic variant (rs7157599, Asn8Ser) in the delta(4)-desaturase, sphingolipid 2 (*DEGS2*) gene. A replication analysis using Caucasian samples showed a directionally consistent trend for cognitive association of a proxy single-nucleotide polymorphism (SNP), rs3783332. Although the *DEGS2* gene is expressed in human brain, it is unknown how *DEGS2* expression varies during human life and whether it is affected by psychiatric disorders and genetic variants. To address these questions, we examined *DEGS2* messenger RNA using next-generation sequencing in postmortem dorsolateral prefrontal cortical tissue from a total of 418 Caucasian samples including patients with schizophrenia, bipolar disorder and major depressive disorder. *DEGS2* is expressed at very low levels prenatally and increases gradually from birth to adolescence and consistently expressed across adulthood. Rs3783332 genotype was significantly associated with the expression across all subjects (F_3,348_=10.79*, P*=1.12 × 10^−^^3^), particularly in control subjects (F_1,87_=13.14, *P*=4.86 × 10^−4^). Similar results were found with rs715799 genotype. The carriers of the risk-associated minor allele at both loci showed significantly lower expression compared with subjects homozygous for the non-risk major allele and this was a consistent finding across all diagnostic groups. *DEGS2* expression showed no association with diagnostic status after correcting for multiple testing (*P*>0.05). Our findings demonstrate that a SNP showing genome-wide association study significant association with cognition in schizophrenia is also associated with regulation of *DEGS2* expression, implicating a molecular mechanism for the clinical association.

## Introduction

Cognitive disability is observed in association with a number of psychiatric disorders.^1, 2^ As there is considerable inter-individual variation in the degree of impairment, it is likely that genetic influences have a role in determining the severity of cognitive deficiency associated with psychiatric disorders. Recently, a genome-wide association study (GWAS) of cognition in Japanese patients with schizophrenia (SCZ) found that impairment was associated with a non-synonymous single-nucleotide polymorphism (SNP, rs7157599, Asn8Ser) in the delta(4)-desaturase, sphingolipid 2 (*DEGS2*) gene.^3^ A replication analysis using Caucasian samples showed a directionally consistent trend for cognitive association of a proxy SNP (rs3783332: *r*^2^=0.76) in high linkage disequilibrium (LD) with rs7157599 (Supplementary Figure 1). The *DEGS2* gene on chromosome 14q32.3 spans 13.3 kb of genomic DNA and contains three exons (the UCSC Genome Browser Human February 2009 (GRCh37/hg19) assembly: chr14:100,612,753–100,626,012). The 323-amino acid protein has a predicted molecular mass of 37.2 kD and shares ~90% similarity with mouse *Degs2*.^4^ *DEGS2* belongs to the desaturase/hydroxylase superfamily and the DEGS2 protein has a sphingolipid dihydroceramide hydroxylase activity. The sphingolipids were first isolated from brain. Sphingomyelin consists of a ceramide with phosphocholine, and is a type of sphingolipid found in animal cell membranes, especially in the membranous myelin sheath that surrounds some nerve cell axons.^5^ Sphingomyelin has roles in signal transduction^6^ and cell apoptosis,^7^ and abnormalities of sphingomyelin can cause several central nervous system diseases, including Niemann–Pick Disease. However, the general function of the *DEGS2* gene in the central nervous system is unclear. This gene is not only expressed in brain, but also in a variety of peripheral tissues, including lung, kidney, intestines and skin.^4^

GWAS of psychiatric disorders and their related clinical phenotypes have identified multiple risk variants.^8, 9, 10^ However, precisely how these SNPs heighten risk is a subject of extensive speculation and relatively little experimentation. Moreover, SNPs from GWAS studies are rarely of known function and are not likely by themselves to be causative. One strategy for identifying a functional association of a genetic locus is to find ‘Expression Quantitative Trait Loci (eQTLs)', which are genomic regions regulating gene expression. SNPs associated with common diseases and phenotypes identified by GWAS are enriched for regulatory regions of the genome,^11, 12^ suggesting that the functional mechanism by which some GWAS variants affect disease and phenotype susceptibility is through gene regulation. The majority of eQTLs are located close to (±1 Mb) the transcription start site of a gene. As some eQTLs appear to be tissue specific,^13^ it is important to perform eQTL analysis in disease-relevant tissues. Dorsolateral prefrontal cortex (DLPFC) is a major component of the high-order associative cortex engaged in attentional and complex cognitive operations,^14, 15^ and dysfunction of this region is prominently related to psychiatric disorders and to the cognitive disabilities associated with them.^16^ We thus focused on DLPFC as a region of human brain to ask whether the risk-associated SNP in *DEGS2* is an eQTL.

To date, although the *DEGS2* gene is expressed in human brain;^4^ the developmental pattern of *DEGS2* expression in the brain and possible alterations in expression in psychiatric disorders has not been examined. Furthermore, the effect of the risk-associated SNP on expression of *DEGS2* also has not been explored. Here, using a large RNA sequencing (RNA-seq) database from postmortem DLPFC of psychiatric patients and control subjects,^17, 18^ we investigated the developmental expression pattern of a *DEGS2* full-length transcript in postmortem human brain, expression of a *DEGS2* transcript in the DLPFC among psychiatric patient cohorts and association of rs3783332, rs7157599 and other SNPs around *DEGS2* with *DEGS2* expression.

## Materials and methods

### Human postmortem brain tissue collection

Postmortem human brain samples from the Brain Tissue Collection of the Clinical Brain Disorders Branch/National Institute of Mental Health (NIMH) and the Lieber Institute for Brain Development were obtained at autopsy. Clinical characterization, neuropathological examination, toxicological analyses, RNA extraction and quality control measures were performed, as described previously.^17^ Each subject had been diagnosed by two board-certified psychiatrists according to the criteria from the *Diagnostic and Statistical Manual of Mental Disorders, Fourth Edition* based on a psychiatric narrative summary compiled from a combination of data from a telephone screening on the day of donation with next-of-kin, police, autopsy and toxicology reports, psychiatric records, family informant interviews with next-of-kin (NIMH psychological autopsy interview and the severe combined immunodeficiency) and/or interviews with psychiatric treatment providers.^18^ Additional postmortem fetal, infant, child and adolescent brain tissue samples (designated University of Maryland cases) were provided by the National Institute of Child Health and Human Development Brain and Tissue Bank for Developmental Disorders (http://medschool.umaryland.edu/BTBank) under contracts NO1-HD-4-3368 and NO1-HD-4-3383. The Institutional Review Board of the University of Maryland at Baltimore and the State of Maryland approved the protocol. The University of Maryland cases were processed, curated, handled and evaluated in a similar fashion to the NIMH and Lieber Institute for Brain Development cases (http://medschool.umaryland.edu/BTBank/ProtocolMethods.html). The messenger RNA (mRNA) expression data from a total of 418 Caucasian postmortem DLPFC gray matter specimens were used for this study. The sample cohort consisted of 96 patients with SCZ (68.8% males, 66 males and 30 females; mean age 46.7±15.6 years), 125 patients with major depressive disorder (58.4% males, 73 males and 52 females; mean age 44.7±14.1 years), 62 patients with bipolar disorder (53.2% males, 33 males and 29 females; mean age 45.9±15.0 years) and 135 healthy subjects (control (CON); 70.4% males, 95 males and 40 females; mean age 33.0±22.0 years). Detailed methods relating to this brain tissue collection have been described elsewhere.^17, 18^ Brain specimens from the Clinical Brain Disorders Branch of the NIMH (JE Kleinman, PI) were transferred under an MTA.

### DNA collection and genotyping

DNA for genotyping was obtained from cerebellar tissues (Qiagen, Valencia, CA, USA), as described previously.^17^ All the brain samples were genotyped using either Illumina Infinium II 650 K, Illumina Infinium HD Gemini 1M Duo or Illumina Human OMNI 5 BeadChips (Illumina, San Diego, CA, USA) according to the manufacturer's instructions. Genotypes were called using Genomestudio software. SNPs were removed if the call rate was <98%, if not in Hardy–Weinberg equilibrium (*P*<0.001) within Caucasian and African American or not polymorphic (MAF <0.01). Imputation was carried out in a cluster server using IMPUTE2 (v2.0.3) software. The reference panels were used from HapMap3 and the 1000 Genomes Pilot Project. We extracted genotype data of 75 SNPs located in or near the *DEGS2* gene (±100 kb), including rs3783332 in Caucasian samples.^3^ Rs3783332 is a proxy for rs7157599 (*r*^*2*^=0.76). SNP rs7157599 was not on the Illumina platform and it did not pass quality control for imputation because of low call rate. Therefore, we genotyped rs7157599 using the TaqMan 5′-exonuclease allelic discrimination assay (Assay ID: C__31234717_10, Applied Biosystems, Carlsbad, CA, USA), as previously described.^19, 20^

### RNA processing and quantification

Processing of postmortem DLPFC gray matter tissue homogenates was as follows.^21^ Poly-A containing mRNA molecules were purified from 1 μg DNase treated total RNA. Following purification, the mRNA was fragmented into small pieces using divalent cations under elevated temperature. Reverse transcriptase and random primers were used to copy the cleaved RNA fragments into single-strand cDNA. Double-stranded cDNA was synthesized using DNA Polymerase I and RNaseH. These cDNA fragments then went through an end-repair process using T4 DNA polymerase, T4 PNK and Klenow DNA polymerase, with the addition of a single ‘A' base using Klenow exo (3′ to 5′ exo minus), then ligated to Illumina PE adaptors using T4 DNA Ligase. An index (upto 12) was inserted into the Illumina adaptors so that multiple samples could be sequenced in one lane of an eight-lane flow cell, if necessary. After processing, these products were then purified and enriched with PCR to create the final cDNA library for high-throughput DNA sequencing using the Illumina HiSeq 2000. The Illumina Real Time Analysis module was used to perform image analysis and base calling, followed by use of BCL Converter (CASAVA v1.8.2) to generate FASTQ files containing sequence reads. Pair-end reads of cDNA sequences obtained by the HiSeq 2000 were aligned back to the human genome reference (UCSC hg19) by splice-read mapper (TopHat v2.0.4),^22^ providing known transcripts from Ensembl Build GRCh37.67. To quantify gene-level expression, we counted the properly paired and mapped reads using htseq-count v0.5.3 (with intersection-strict mode), and RPKM (Reads Per Kilobase per Million mapped reads) was calculated.

### The quality control procedure for the RNA-seq

Outliers due to measurement errors were omitted if subject had a value more than three times the interquartile range from either 25th percentile or 75th percentile. Of 418 subjects, 16 samples were outliers (3.8%) and were excluded from this study. Developmental expression pattern was investigated on the 128 nonpsychiatric controls from fetal life to advanced age. Further expression analyses were performed on the 366 subjects with age >16 and RNA integrity number >7. Detailed demographic information of 366 subjects included in these expression analyses is shown in Supplementary Table 1.

### Statistical analyses

The effects of diagnosis on the *DEGS2* expression were analyzed by analyses of covariance with diagnostic status as an independent variable and age, sex and RNA integrity number as covariates using PASW Statistics 18.0 software (SPSS Japan, Tokyo, Japan). The effects of *DEGS2* genotypes and diagnosis–genotype interaction on the *DEGS2* expression were analyzed by analyses of covariance with each genotype and diagnostic status as independent variables and age, sex and RNA integrity number as covariates. *Post hoc* tests with Fisher's least significant difference were used to evaluate significant differences among diagnostic groups or genotype groups.

### *In silico* analysis

PolyPhen2 is a tool, which predicts the possible impact of an amino acid substitution on the structure and function of a human protein using straightforward physical and comparative considerations.^23^ SNPs3D is a website, which assigns molecular functional effects of nsSNP on the basis of structure and sequence analysis.^24^ A positive Support Vector Machine score in SNPs3D indicates a variant classified as non-deleterious and a negative score indicates a deleterious case. Larger scores are more confident. Accuracy is significantly higher for scores >0.5 or <−0.5. AliBaba 2.1 is a program for predicting binding sites of transcription factors in a sequence using binding sites from TRANSFAC Public (http://www.gene-regulation.com/pub/programs/alibaba2/index.html).

## Results

### Developmental expression pattern of a DEGS2 transcript in postmortem brains

**Figure 1:**
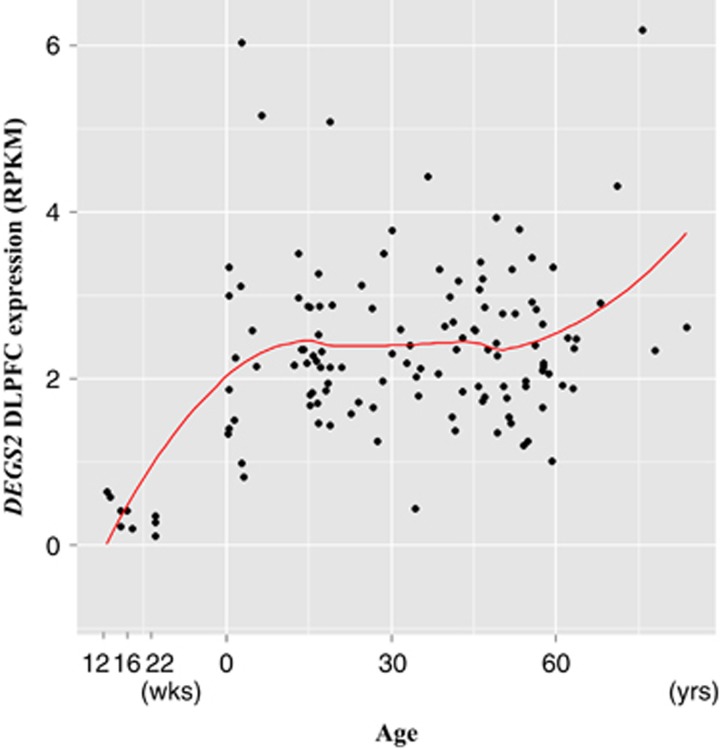
Developmental expression pattern of a *DEGS2* transcript in postmortem brains. The expression of *DEGS2* in the DLPFC across the lifespan was displayed from gestational week 12 through 22 and from birth through old age. Gene expression in each individual subject is shown as a black dot. A curved line represented a LOESS fit across the lifespan. DLPFC, dorsolateral prefrontal cortex; RPKM, Reads Per Kilobase per Million mapped reads.

To investigate the developmental profile of expression of *DEGS2*, we used samples from the DLPFC in nonpsychiatric controls across the human lifespan including during fetal life. *DEGS2* is expressed at very low levels during the prenatal period and increases gradually from birth to adolescence and then remains at almost constant levels until older age and increases again (Figure 1).

### DEGS2 expression in postmortem DLPFC in selected psychiatric disorders

**Figure 2:**
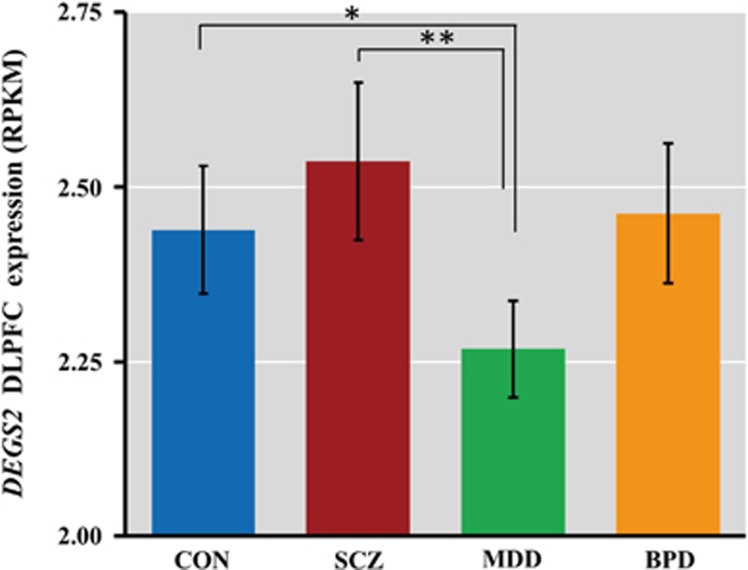
*DEGS2* expression in postmortem DLPFC across several psychiatric disorders. *P=0.037, **P=0.017. DLPFC, dorsolateral prefrontal cortex; RPKM, Reads Per Kilobase per Million mapped reads.

We found a marginal association of diagnosis with *DEGS2* DLPFC expression (F_3,359_=2.39, *P*=0.069, Figure 2). *DEGS2* expression was decreased in major depressive disorder compared with CON (*post hoc* with Fisher's least significant difference; *P*=0.037) and SCZ (*post hoc* with Fisher's least significant difference; *P*=0.017). There were no significant differences in expression between SCZ and CON or between bipolar disorder and other diagnosis (*P*>0.05). None of these associations, however, are statistically significant after Bonferroni correction (0.05/6=0.008). As these differences in expression among diagnosis status may be affected by sample size and potential patient-related confounding factors, such as medications, the data suggest that further research using larger sample sizes may be fruitful.

### Association of rs3783332 genotype with DEGS2 expression

**Figure 3:**
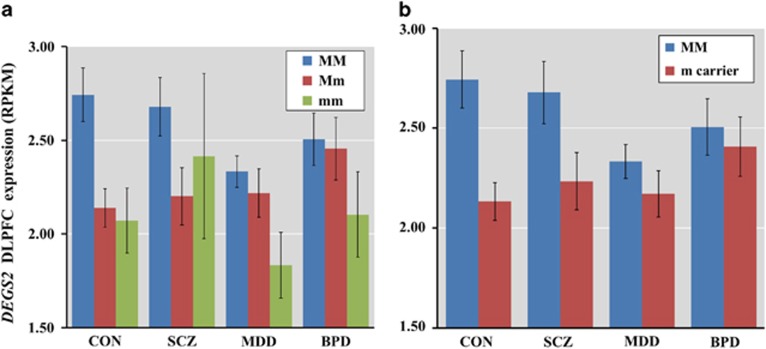
Association of rs3783332 genotype on *DEGS2* expression. (**a**) Additive association of rs3783332 on the *DEGS2* expression. MM >Mm *P*=0.0031, MM >mm *P*=0.033. (**b**) Dominant model association of rs3783332 on the *DEGS2* expression. Means±s.e. are shown. DLPFC, dorsolateral prefrontal cortex; M, major allele; m, minor allele; RPKM, Reads Per Kilobase per Million mapped reads.

We next examined whether the clinically significant SNP at rs3783332 is associated with *DEGS2* expression in human brain in the Caucasian sample. Rs3783332 genotype was significantly associated with expression in DLPFC, using additive (F_6,344_=5.68, *P*=0.0037, Figure 3a) and dominant models (F_3,348_=10.79*, P*=0.0011, Figure 3b). The carriers of the risk-associated minor allele, associated with greater cognitive impairment, showed lower expression compared with subjects homozygous for the non-risk major allele and this was a consistent finding across all diagnostic groups. There were no significant diagnosis–genotype interactions (*P*>0.05).

**Figure 4:**
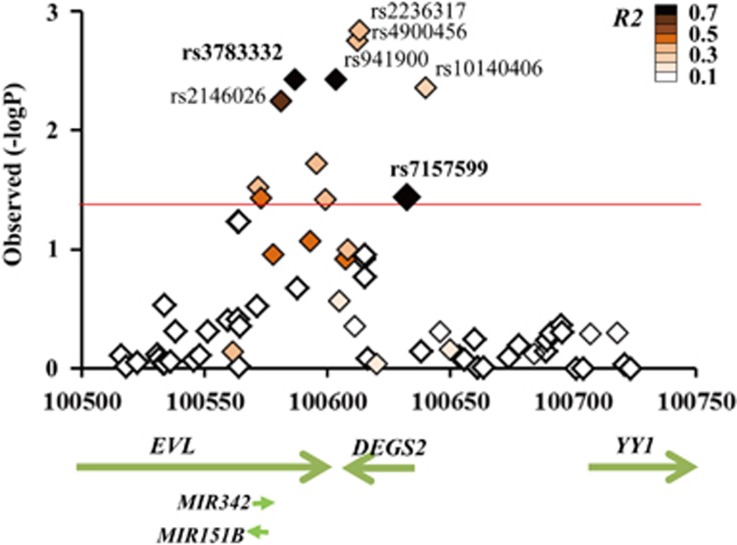
Association of SNPs around *DEGS2* on *DEGS2* expression. *P*-values (−log_10_) of additive effect of each SNP on *DEGS2* expression are shown in regions peripheral to the *DEGS2* gene. *R*^*2*^ scores between rs7157599 and each SNP in CEU population (HapMap3, release 2) are represented with increasing color intensity, as shown by color bars. The red line indicates a *P*-value of 0.05. SNP, single-nucleotide polymorphism.

To find additional eQTLs, we investigated the interaction between genotypes and expression for 76 SNPs including rs7157599 around the *DEGS2* gene (±100 kb) in the combined group of subjects (Figure 4). Several SNPs in LD with rs7157599 as well as rs7157599 itself (*P*=0.037) were also associated with the *DEGS2* expression (rs2236317 had the most significant *P*=0.0015). These associations are strongest in the CON sample (Supplementary Table 2 and Supplementary Figures 2–4). The SNP at rs3783332 showed the most significant association with the expression in the CON sample (additive effect of rs3783332 genotype on the expression, F_2,86_=6.50, *P*=0.0023; dominant effect, F_1,87_=13.14, *P*=0.00049).

To examine the possibility that this missense polymorphism (rs7157599) might alter protein structure, *in silico* analysis was performed using the PolyPhen2 and the SNPs3D. Polyphen2 predicted that the impact of the rs7157599 was benign. The Support Vector Machine score in SNPs3D was 2.91, indicating that this variant was non-deleterious for protein structure. Though rs7157599 is a missense polymorphism (nsSNP), these *in silico* results suggest, but do not establish, that this particular amino acid substitution may not have a functional impact on *DEGS2* protein structure. Next, we examined whether transcription factor binding sites might be altered by rs7157599 and the most significant eQTLs, rs2236317 and rs3783332, in the combined sample and in CON, respectively. rs3783332 was identified as possible influencing regulation of gene transcription, whereas rs7157599 and rs2236317 were not found to be related to gene regulation. An Sp-1 binding site was altered by a single-nucleotide change of rs3783332; the sequence CCTTCT**C**TTC (Major C allele is a non-risk allele for cognitive impairment) is an Sp-1 binding site, whereas the sequence CCTTCT**T**TTC disrupts this Sp-1 binding site. These findings suggest that the SNP at rs3783332 might be a functional variant and this alteration could lead to dysregulation of the transcriptional activity of the *DEGS2*.

## Discussion

In addition to earlier evidence for a clinical relationship between *DEGS2* polymorphisms (rs7157599 and rs3783332) and cognitive deficits in patients with SCZ,^3^ here we have demonstrated a significant association between the same *DEGS2* polymorphisms and *DEGS2* expression in human brain. The *DEGS2* minor allele at these SNPs related to cognitive impairment was associated with decreased expression in normal controls and three independent diagnostic groups. Ten SNPs in low-to-high LD (*r*^*2*^=0.26–0.75) with rs7157599 (related to cognitive impairment) were also associated with *DEGS2* expression (*P*<0.05). In contrast, independent SNPs (*r*^*2*^=0) showed no association with *DEGS2* expression (*P*>0.05). Bioinformatic analysis suggested that the missense polymorphism rs7157599 did not alter protein structure but the proxy variant rs3783332 of rs7157599 was associated with the regulation of gene transcription. These findings suggest that the positive GWAS signal might contribute to cognitive impairment through regulation of *DEGS2* expression in human brain.

The trait/disease-associated SNPs identified by GWAS have been significantly overrepresented in regions of regulatory genetic elements compared with the SNPs randomly selected from the genotyping arrays.^25^ In addition, the SNPs in close proximity to genes from all positive GWAS signals seem to explain more variation of the examined phenotypes and to replicate at higher rates compared with intergenic SNPs.^26, 27^ eQTL analysis is one way to begin to examine the molecular signature of findings from GWAS.^13^ For example, one study showed ~20% overlaps between *cis*-eQTL signals in human brain and GWAS hit signals for adult-onset neurological disorders. We show that for *DEGS2*, the detected signal by GWAS was related to a human brain eQTL. To confirm whether our detected association of the *DEGS2* polymorphism did not derive from a relationship between the polymorphisms and genes other than *DEGS2*, we further screened the relationship between the genetic variant rs3783332 on expression of several genes in close proximity to *DEGS2* (±1 Mb) in our Caucasian control samples. Of genes expressed moderately to highly in DLPFC, the variant rs3783332 was related only to *DEGS2* expression, in the region around this gene (data not shown).

We explored the *DEGS2* expression pattern during human prefrontal cortical development and demonstrated that the *DEGS2* gene was most abundantly expressed in DLPFC after birth. *DEGS2* encodes a bi-functional enzyme that can act as both a sphingolipid delta(4)-desaturase and as a sphingolipid C4-hydroxylase.^4^ This enzyme is involved in the biosynthesis of phytosphingolipids in phytosphingolipid-containing tissues, such as skin, intestines and kidney. Sphingolipids are ubiquitous components of the plasma membrane in all animals, and consist of an obligatory sphingoid base (phytosphingosine, sphingosine or others). The phytosphingosine is a minor sphingoid base in mammalian cells.^28^ Phytoceramide is a fatty acid derivative of phytosphingosine, and is a common backbone of complex phyto-type sphingolipids. As the ceramide structure varies due to varying hydroxylation and desaturation in the sphingoid base, the *DEGS2* gene has a role in maintaining the ceramide structure. *Degs2* knockout mice lack both phytoceramide and phytosphingosine in intestines and kidney.^29, 30^ The expression pattern of *DEGS2* correlates with the distribution of phytosphingolipids.^4, 31^ In addition, since expression of *DEGS2* mRNA regulates synthesis of phytosphingolipids during keratinocyte differentiation,^4^ the risk *DEGS2* polymorphism may be associated with lower synthesis of sphingolipids in brain. There have been no studies of brain structure or function in *Degs2* knockout mice or where phytosphingosine is located in neurons. Further research is needed to clarify the role of phytosphingolipids in signal transduction and cell apoptosis in the brain similar to sphingomyelin and how the decreased synthesis of phytosphingolipids in the central nervous system contributes to pathogenesis of cognitive impairments.

We found effects of genetic variants on *DEGS2* expression in our total sample. To reduce the possibility of artifactual association owing to diagnostic stratification, we performed subgroup analyses with and without control samples. When dividing these subjects into patient and control groups, the signficant genotype effects were apparent primarily in the control group (Supplementary Figures 2–4) although the directions of the association were consistent in all the samples. Rs3783332 was the most strongly associated variant for *DEGS2* expression in the control group. These associations were weaker in patient samples possibly owing to the influence of patient-specific confounds, for example, medications and duration of illness. To obtain more relevant genotype effect in patient groups, much larger sample sizes would be needed.

We found that several SNPs in low-to-high LD with the rs7157599 were associated with *DEGS2* expression. As these SNPs with stronger association with mRNA expression than rs3783332 (which was associated with cognitive deficits in Caucasians) might also affect cognitive deficits, we further examined the associations between the top five eQTLs (rs2236317, rs4900456, rs941900, rs10140406 and rs2146026) and cognitive deficits in our Caucasian sample, as previously described.^3^ Of the five SNPs, rs2146026 and rs941900, which are in high LD with the rs7157599, showed directionally consistent trend associations with cognitive deficits (*P*<0.05), as expected. Interestingly, *in silico* analysis using AliBaba 2.1 showed that both SNPs were also associated with changes of transcription factor binding sites (data not shown). Taken together, the three SNPs rs3783332, rs2146026 and rs941900 may all be functional variants related to *DEGS2* expression. To confirm whether a single-nucleotide change of these SNPs truly contributes to change of transcription factor binding site, further research is required.

There are some limitations to this study that merit discussion. Although we have identified rs7157599 in the *DEGS2* by GWAS of cognitive deficits in SCZ,^3^ the top hit SNP in that study was just shy of genome-wide significance (*P*=5.0 × 10^−^^8^). Validation of these findings will require study in a larger sample. We measured *DEGS2* expression in human brain homogenates. The use of homogenates makes it impossible to distinguish whether our findings are present in neurons, glia or both. Clarification of the cell type related to this variant could provide further information underlying the genomic mechanism for cognitive impairment. In addition, genetic variants in the *DEGS2* region have not been associated with SCZ in the latest GWAS from a largely European sample, although genetic variants including rs2693698 in the *BCL11B* region, which sit ~890 kb away from the *DEGS2* region have been associated with this disorder.^8^ The genome-wide significant SNP at rs2693698 in the *BCL11B* was not associated with the *DEGS2* expression, whereas rs3783332 also was not associated with the *BCL11B* expression in DLPFC. To summarize these findings, our identified association between rs3783332 and *DEGS2* expression might contribute to susceptibility to cognitive impairment but not SCZ itself.

In conclusion, our findings demonstrate that a *DEGS2* polymorphism associated with cognition in SCZ is also associated with *DEGS2* expression in DLPFC. We suggest that this variant may have a role in the cognitive impairments noted in psychiatric disorders through genetic control of the *DEGS2* expression. Identification of the biological effects of this gene on the brain may help to reveal a molecular mechanism for the clinical association involved in our studies.
