# Supplementary material for: DEGS2 polymorphism associated with cognition in schizophrenia is associated with gene expression in brain
Source: Transl Psychiatry. 2015 Apr 14;5(4):e550–. doi: 10.1038/tp.2015.45 (PMC4462608; doi:10.1038/tp.2015.45)
Supplement: Supplementary Information [file tp201545x1.doc]

**Supplementary Information**

***DEGS2* polymorphism associated with cognition in schizophrenia is associated with gene expression in brain**

Kazutaka Ohi^1,2^, Gianluca Ursini^1^, Ming Li^1^, Joo Heon Shin^1^, Tianzhang Ye^1^, Qiang Chen^1^, Ran Tao^1^, Joel E. Kleinman^1^, Thomas M. Hyde^1,3,4^, Ryota Hashimoto^2,5^and Daniel R. Weinberger^1,3,4,6,7^

*^1^Lieber Institute for Brain Development, Johns Hopkins Medical Campus, Baltimore, MD, USA;*

*^2^Department of Psychiatry, Osaka University Graduate School of Medicine, Osaka, Japan;*

*^3^Department of Psychiatry, Johns Hopkins University School of Medicine, Baltimore, MD, USA;*

*^4^Department of Neurology, Johns Hopkins University School of Medicine, Baltimore, MD, USA;*

*^5^Molecular Research Center for Children's Mental Development, United Graduate School of Child Development, Osaka University, Osaka, Japan;*

*^6^Department of Neuroscience, Johns Hopkins University School of Medicine, Baltimore, MD, USA;*

*^7^Institute of Genetic Medicine, Johns Hopkins University School of Medicine, Baltimore, MD, USA.*

**Supplementary information content**

Supplementary Figures 1-4 and Supplementary Tables 1-2.

**
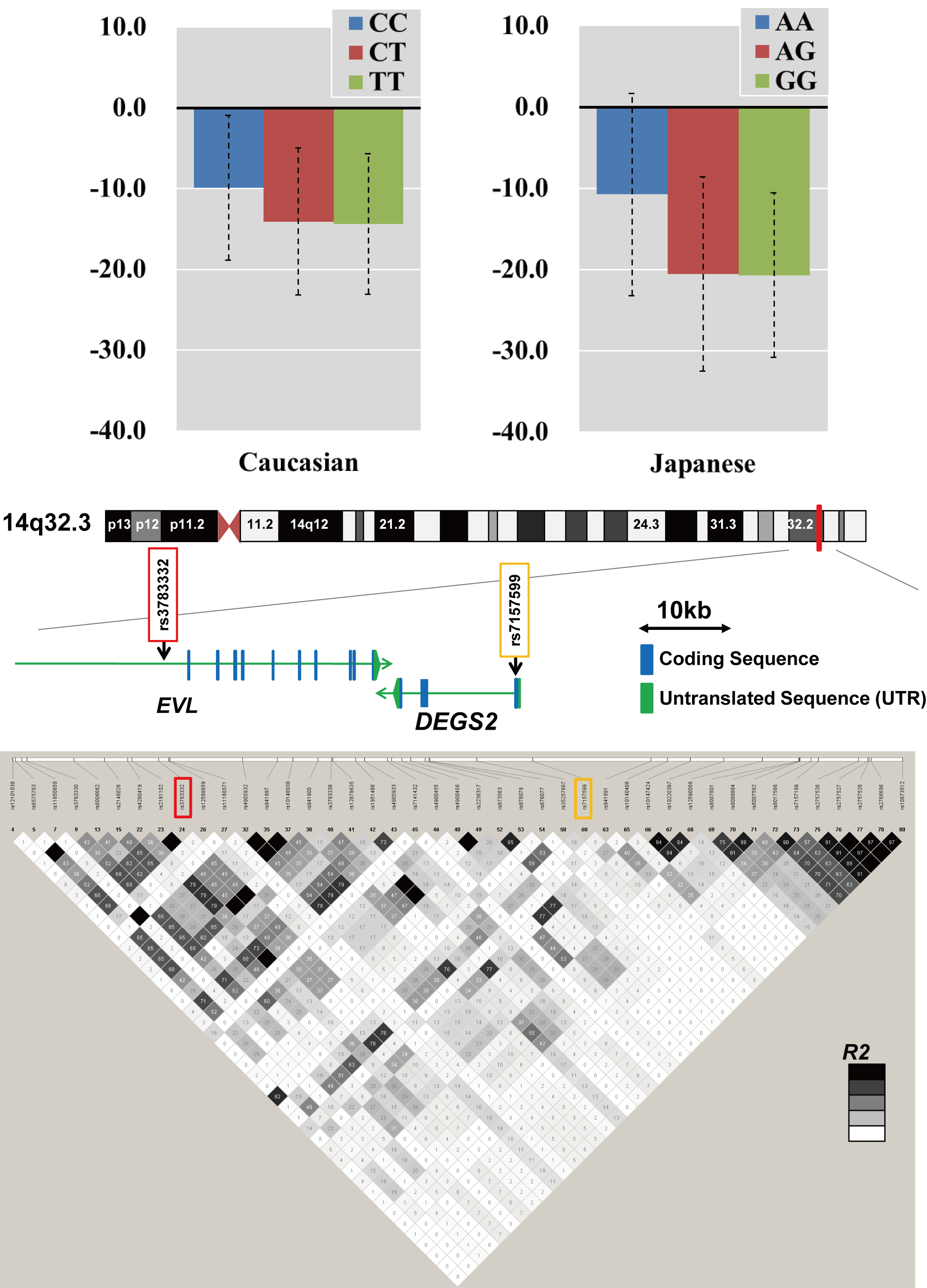
**

**Supplementary Figure 1.** Associations between two SNPs (rs378332 and rs7157599) and cognitive deficit and genomic structure around *DEGS2* gene.

According to previous results (Hashimoto *et al.*, 2013), the associations of two SNPs with cognition in Caucasian (*P*=0.03) and Japanese (*P*=5.39×10^-7^) samples are shown. Means ± SD are shown. Based the UCSC Genome Browser Human Feb. 2009 (GRCh37/hg19) assembly, the genomic structure around *DEGS2* is shown. The locations of rs3783332 and rs7157599 are indicated by arrows. LD between pairwise SNPs are shown using *R2* values at the bottom of the map of the gene structure for the HapMap CEU samples (HapMap Data Rel 28 PhaseII+III, August10, on NCBI B36 assembly, dbSNP b126). High levels of LD are represented by black coloring with increasing color intensity, as shown by color bars. The rs3783332 is the SNP strongest LD with rs7157599 in this region.

**Supplementary Figure 2.** Effect of rs3783332 genotype on the *DEGS2* expression within the control group.

**(a)** Additive effect: Genotype effect; *F_2,86_*=6.50, *P*=0.0023, **(b)** Dominant effect: Genotype effect; *F_1,87_*=13.14, *P*=0.00049. Means ± SE are shown.

**Supplementary Figure 3.** Effect of rs3783332 genotype on the *DEGS2* expression within patient group.

**(a)** Additive effect: Genotype effect; *F_2,255_*=1.95*, P*=0.14, **(b)** Dominant effect: Genotype effect; *F_1,258_*=3.34, *P*=0.069. Diagnostic status, age, sex and RIN were included as covariates in ANCOVA analysis.

**
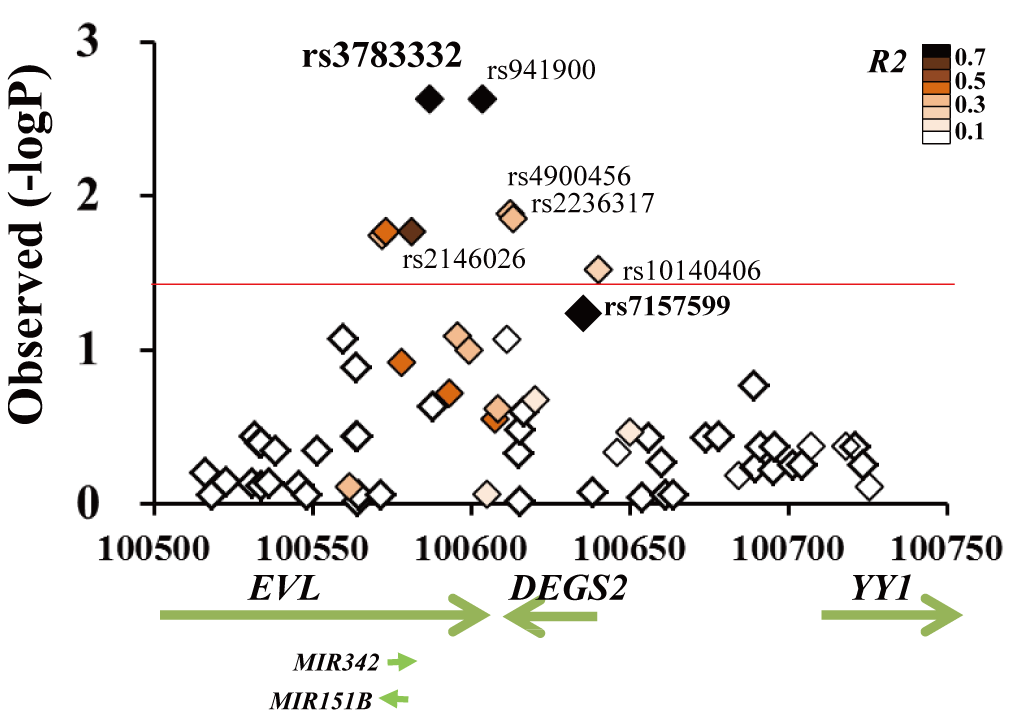
**

**Supplementary Figure 4.** Additive effects of SNPs around *DEGS2* gene on *DEGS2* DLPFC expression within control samples.

*R^2^* scores between rs7157599 and each SNP in CEU population (HapMap 3, release 2) are represented with increasing color intensity, as shown by color bars. The SNP at rs3783332 was most strongly associated with the *DEGS2* expression (*P*=0.0023, Supplementary Figure 2a).

**Supplementary Table 1. Demographic information of patients with schizophrenia, major depression disorder and bipolar disorder and healthy subjects**

|  | SCZ | MDD | BPD | CON |
| --- | --- | --- | --- | --- |
| Variable | (*N*= 93) | (*N*= 122) | (*N*= 58) | (*N*= 93) |
| Age (years) | 46.5 ± 15.8 | 44.9 ± 13.9 | 45.4 ± 14.8 | 42.7 ± 16.2 |
| Gender (male/female) | 66/27 | 72/50 | 30/28 | 73/20 |
| RIN | 8.02 ± 0.82 | 8.07 ± 0.87 | 7.80 ± 1.03 | 8.45 ± 0.52 |

RIN, RNA integrity number. The mean ± SD are shown.

**Supplementary Table 2. Each genotypic distribution of rs3783332 for cases and controls**

|  | Cases (*N*=267) | | |  | Controls (*N*=92) | | |
| --- | --- | --- | --- | --- | --- | --- | --- |
|  | mm | Mm | MM |  | mm | Mm | MM |
| Variables | (*N*=15) | (*N*=97) | (*N*=155) |  | (*N*=4) | (*N*=41) | (*N*=47) |
| Age (years) | 43.8 ± 12.7 | 44.1 ± 14.0 | 46.6 ± 15.3 |  | 53.1 ± 7.0 | 43.9 ± 16.3 | 40.8 ± 16.5 |
| Gender (male/female) | 10/5 | 61/36 | 92/63 |  | 4/0 | 33/8 | 35/12 |
| RIN | 8.03 ± 0.79 | 7.93 ± 0.96 | 8.03 ± 0.85 |  | 8.25 ± 0.58 | 8.46 ± 0.54 | 8.46 ± 0.51 |

M; major allele, m; minor allele. The mean ± SD are shown.
